# Seed color represents salt resistance of alfalfa seeds (*Medicago sativa* L.): Based on the analysis of germination characteristics, seedling growth and seed traits

**DOI:** 10.3389/fpls.2023.1104948

**Published:** 2023-02-16

**Authors:** Jin Xie, Yao Li, Gaoqian Jiang, Hongyong Sun, Xiaojing Liu, Lipu Han

**Affiliations:** ^1^ Key Laboratory of Agricultural Water Resources, Institute of Genetics and Developmental Biology, Chinese Academy of Sciences, Shijiazhuang, Hebei, China; ^2^ Hebei Key Laboratory of Soil Ecology, Institute of Genetics and Developmental Biology, Chinese Academy of Sciences, Shijiazhuang, Hebei, China; ^3^ College of Advanced Agricultural Sciences, University of Chinese Academy of Sciences, Beijing, China

**Keywords:** seed color, germination, seedling growth, endogenous hormone, seed coat

## Abstract

**Introduction:**

Alfalfa (*Medicago sativa* L.) has significant feed value and ecological improvement function of marginal land. The difference in the maturity period of seeds in the same lots may be a mechanism of environmental adaptation. Seed color is a morphological trait associated with seed maturity. A clear understanding of the relationship between the seed color and seed stress resistance is helpful for seed selection for marginal land.

**Methods:**

This study evaluated the seed germination parameters (germinability and final germination percentage) and subsequent seedling growth (sprout height, root length, fresh and dry weight) of alfalfa under different salt stress, measured the electrical conductivity, water uptake, seed coat thickness, and endogenous hormone content in alfalfa seeds with different colors (green, yellow and brown).

**Results:**

The results showed that seed color significantly influenced the seed germination and seedling growth. The germination parameters and seedling performance of brown seeds were significantly lower than that of green and yellow seeds under different salt stress. The germination parameters and seedling growth of brown seed declined most obviously with the aggravation of salt stress. The results suggested that brown seeds were less resistant to salt stress. Seed color had a significant effect on electrical conductivity, and it indicated that the yellow seeds had higher vigor. The seed coat thickness of various colors did not differ significantly. The seed water uptake rate and hormone content (IAA, GA3, ABA) in brown seeds were higher than that in green and yellow seeds, while the (IAA+GA3)/ ABA in yellow seeds were higher than green and brown seeds. The alterations in seed germination and seedling performance among seed colors are likely due to the combination effect of the content and balance between IAA+GA3 and ABA.

**Discussion:**

These results could improve the understanding of stress adaptation mechanisms of alfalfa and provide a theoretical basis for screening alfalfa seeds with high stress resistance.

## 1 Introduction

Alfalfa (*Medicago sativa* L.) is one of the most prevailing cultivated forage crops in the world, with a global planting area of 35 million hm^2^ (Radovic et al., 2009). Alfalfa is not only endowed with high nutrient content and excellent palatability for livestock but also has a high value to soil improvement due to the deep-root system and nitrogen-fixing ability (Singer et al., 2018). Therefore, planting alfalfa on marginal land like saline-alkali areas is proposed as a method to decrease soil salinization and increase the utilization of saline-alkali land (Monirifar et al., 2020). For instance, the alfalfa industry in China is booming with the implementation of the “Grass-based Livestock Husbandry” and “Grain to Feed” policy (Mao et al., 2016), but limited by the scarcity of arable land in China, planting alfalfa on saline-alkali soil become an important strategy for national food security (Xia et al., 2018; Cao et al., 2021).

Successful seed germination is the beginning of the plant life cycle. However, seed germination is highly restricted by the environment (e.g. salt, water, and temperature) (Gul et al., 2013; Zhang et al., 2015). Seed heteromorphism is considered an adaptive strategy that helps plants to ensure seed germination and seedling establishment in natural conditions with spatial-temporal variability (Xu et al., 2011; Gul et al., 2013; Wang et al., 2015; Liu et al., 2018). Different seed color of alfalfa is a type of heteromorphism controlled by maturity (Rahman and McVetty, 2011; Velijević et al., 2017). The diverse ripening time provides a strategy for seeds to spread germination across time to reduce the risk of premature death during unfavorable environmental conditions (Bewley et al., 2013; Porter, 2013). Previous researches have reported that seed color affects seed vigor and germination (Liu et al., 2007; Mavi, 2010; Attri et al., 2021), but the relationship between seed vigor and seed color varied with species. For example, red clover (*Trifolium pratense*) seeds with light colour had stronger vigor than dark and mixed color (Velijević et al., 2017), the light-colored horse gram (*Macrotyloma uniflorum*) seeds showed the highest germination percentage followed by medium and dark colored seeds (Singh et al., 2009); while black guar (*Cyamopsis tetragonoloba* (L.) Taub) seeds had higher germination than dull-white-colored seeds (Liu et al., 2007). However, fewer studies have been undertaken to analyze the effects of seed color on seed vigor and germination of alfalfa (Stewart and Carlson, 1932; Lv et al., 2021; Liu et al., 2022), and whether the alfalfa seeds of different colors have specific resistance to stress (e.g. salt stress) needs further study.

The morphological traits of seed coat contribute to the germination mechanisms (Nonogaki, 2019; Kimura et al., 2020). Seed coat creates a special barrier to water to tissues and pose a problem for imbibition (Kelly et al., 1992; Gardarin et al., 2010). It reported that large volumes of water for germination was uptake during the imbibition period (Souza and Marcos-Filho, 2001; Srii et al., 2022). Research suggested that the cuticle and palisade layers in seed coat are important factors causing impermeability (Noodén et al., 1985; Souza and Marcos-Filho, 2001; Bewley et al., 2013). Seeds with thick cuticles and palisade layers slow down the rate of water uptake (Mcdonald et al., 1988). Seed coat thickness may vary with seed color (Coen and Magnani, 2018), so the change of germination among different seed colors may due to the permeability properties of seed coat. But there is less knowledge about relationship between seed color, seed coat permeability properties and seed coat thickness.

Hormone interactions can effectively regulate seed germination and promote seedling growth (Shu et al., 2016; Liu et al., 2022). Indoleacetic acid (IAA) and gibberellin (GA) are critical germination promoters, while abscisic acid (ABA) is a germination inhibitor (Ravindran and Kumar, 2019; Chen et al., 2020). The content of hormones varied during seed ripening (Miransari and Smith, 2014; Bosco et al., 2015; Tang et al., 2022), however, few studies have determined the difference of hormones in alfalfa seeds of different coat colors (Wang et al., 2015; Liu et al., 2018). In addition, hormones are involved in seed response to environmental stress (Popko et al., 2010; Miransari and Smith, 2014). The accumulation of hormones in different colored seeds may be a protective mechanism to avoid seedling death due to unfavorable conditions (Baskin et al., 2000). Making clear the effect of hormone content on the stress resistance of alfalfa seeds provides promising method for improving seed germination.

In this study, we seek to better understand whether salt resistance of alfalfa seeds affected by seed color, and the role of seed coat and hormones in controlling the salt resistance. To do so, we investigate the difference of seed germination performance (germinability, final germination percentage) and seedling growth (sprout height, root length, fresh and dry weight) among seed color and salt stress. We further detected the seed vigor, water uptake rate, seed coat thickness and hormone content (GA3, IAA and ABA). We hypothesize that 1) salt resistance of alfalfa seeds increased with the deepen of seed color (i.e. brown>yellow>green); 2) the endogenous hormone played more important role in regulating the salt resistance than seed coat permeability properties. These study could help reveal the adaptation strategies of seeds to environmental stress and provide a theoretical basis for screening alfalfa seeds with high salt tolerance in China.

## 2 Materials and methods

### 2.1 Experiment materials

The alfalfa seed (*Alfalfa cultivar* ‘Zhongmu No. 3’) was provided by a specialized seed producer (Xintai Zhouquan Agricultural Technology Co., LTD, China). The similar size of healthy and plump seeds was screened and then sorted into three groups (brown, yellow and green) according to the seed coat color by visual inspection. The seed proportion was calculated by counting the number of seeds with different colors. Yellow seeds comprised the major part (59%) of the seed lot used for this study, while the relative amount of brown seeds was 33%. Green seeds comprised only 8% of the seed lot (**Table 1**). GA3, D-IAA, D-ABA, D-GA4 were purchased from OlChemIm Ltd. (Olomouc, Czech Republic). IAA was purchased from LGC Standards Ltd. (Tetdington, UK). ABA was purchased from Sigma-Aldrich (Merck, Germany).

**Table 1:** Seed characters of different coat color.

Coat color	Percentage (%)	Thousand seed weight (g)
Mix	100	2.082 ± 0.064
Brown	33	2.167 ± 0.049
Yellow	59	2.103 ± 0.039
Green	8	2.048 ± 0.034

### 2.2 Seed germination experiment

The alfalfa seeds were sterilized with 75% alcohol for 30 min, rinsed with distilled water three times to remove the remaining alcohol, and then soaked in distilled water. Thirty soaked seeds of different colors were incubated on double-layered filter paper that was placed in each 12-cm-diameter Petri dish with 8 mL solutions, i.e. CK (0.00 mol/L NaCl solution), 0.05 mol/L NaCl solution, 0.10 mol/L NaCl solution, 0.15 mol/L NaCl solution, and 0.20 mol/L NaCl solution. All experiments were performed with six replicates. Petri dishes were placed in the controlled climatic chamber with a photoperiod of 12 h/12 h (day/night). The temperature was set to 25°C, the light was set to 5000lx, and relative humidity was set to 65% in day time, while they were set to 20°C, 0lx and 65% at night time. Seeds were provided with additional solutions separately to prevent drying. Germinated seeds (as judged by the appearance of a visible 1 mm radicle) were counted every day until the number stopped increasing, and finally, the germination test lasted for 7 days. On the seventh day, the sprout and root length of seedlings in each petri dish were measured. For those petri dishes with more than 20 seeds germinated, the sprout height and root length of 20 randomly selected seedlings were measured, followed by seedling fresh and dry weight measurement. For those petri dishes with less than 20 seeds germinated, all the seedlings in the petri dish were measured. The germinability and final germination rate were estimated to describe the influence of seed color and salt stress on the germination process.

The germinability (*Ga*) refers to the total germination on which day the daily germinated seed reaches its peak. Based on the pre-experiment, the germination peak of alfalfa seeds was on the 3^rd^ day in this study, so it was calculated as:


$$Ga=\frac{germinated\;seeds\;on\;the\;third\;day}{total\;seeds}\times 100\%$$

e final germination (*Gf*) rate was calculated as:


$$Gf=\frac{germinated\;seeds\;on\;the\;seventh\;day}{total\;seeds}\times 100\%$$

### 2.3 Seed traits

#### 2.3.1 Electrical conductivity tests

Electrical conductivity tests were performed to determine seed vigor. One hundred seeds of different colors were randomly selected and weighed with a precision electronic scale (0.0001g, PWN124ZH/E, OHAUS, USA). The seeds were placed in a beaker filled with 80mL deionized water, covered with film and cultured in a 25°C chamber for 24h. Each sample was replicated four times (100 seeds × 3 color × 4 replicates). The electrical conductivity of deionized water and leachates in each beaker was measured by using conductivity meter (DDS-307A, INESA (Group) Co., Ltd.) every hour. The electrical conductivity was calculated as:


$$EC\left[\mu S/\left(cm\cdot g\right)\right]=\left(EC_{1}-EC_{0}\right)/W$$

ere *EC_1_* = conductivity of each sample at the different soaking time;

*EC_0_* = conductivity of deionized water only;

*W*= the weight of one hundred seeds of each sample.

#### 2.3.2 Seed imbibition test

Seed imbibition was measured by the changes in seed volume and weight. During the imbibition, twenty seeds of each color were placed on the wetting filter papers, and then put into climatic chamber with the temperature, light, and moisture set at 25°C, 0lx, and 65% for 24h. Every one hour, the seeds were taken out in sequence and the seed surface were dried with paper quickly. Then the seed weight was measured by a precision electronic balance (0.0001g, PWN124ZH/E, OHAUS, USA). The seed length (L), width (WD), and thickness (T) were measured by an electronic digital indicator. The seed volume (*SV*) was calculated using the formula given below:


$$SV\left(mm^{3}\right)=\left(\pi \times L\times WD\times T\right)/6$$

ere L = seed length; *WD* = seed width; *T*= seed thickness.

#### 2.3.3 Seed coat thickness

To determine the seed coat thickness, the alfalfa seed was crosscut and viewed using Scanning Electron Microscope (Zeiss Gemini 300). The coat thickness of three points for each seed was estimated, and the measurement point were on back of seed and ventral of seed. Five seeds were used for each color in this test.

#### 2.3.4 Seed endogenous hormone

The content of Gibberellin (GA3), indoleacetic acid (IAA) and Abscisic Acid (ABA) in seeds of different colors were determined by High-Performance Liquid Chromatography (HPLC)(Agilent-1290, Agilent Technologies, USA) coupled to a mass spectrometer (MS/MS)(AB Sciex-6500 Qtrap, Allen-Bradley, USA). Each sample was replicated four times (3 colors × 4 replicates). The brief process was that 1.5g seed material was ground in liquid nitrogen, followed by adding mixed solution of isopropyl alcohol, water and hydrochloric acid. Then the specimen was shaken for 30 minutes respectively after adding standard solution and methylene chloride. Next, the specimen was centrifuged (13000 r/min) for 5 minutes to gain organic substance. Next, the organic substance was dried using Termovap Sample Concentrator (NDK200-2N, Hangzhou Miu Instruments Co., Ltd, China) and redissolved by methyl alcohol. After that, the solution was centrifuged again (13000 r/min) for 10 minutes and then filtrated. The whole solvent extraction was operated at 4 °C. Finally, the hormones were measured by HPLC-MS/MS.

### 2.4 Statistical analysis

Before analysis, all variables were tested for normal distribution by Kolmogorov-Smirnov’s test, the homogeneity of variances was examined by Levene’s test, and the final germination and the sprout length of yellow seeds were log-transformed to meet the require of homogeneity. The statistical significance of seed color on seed traits, seed germination process and seedling growth were evaluated using one-way analysis of variance (ANOVA) and least significance difference (LSD) test. For data that did not conform to homogeneity of variances after transformation, Mann-Whitney U test was used to evaluate the effects of seed color. The analyses were performed with IBM SPSS 22.0 (IBM Corp., Armonk, NY, USA), and significance was examined at a level of *P*<0.05. All data are presented as mean ± standard error (SE).

## 3 Results

### 3.1 Seed germination and seedling growth

**Figure 1:**
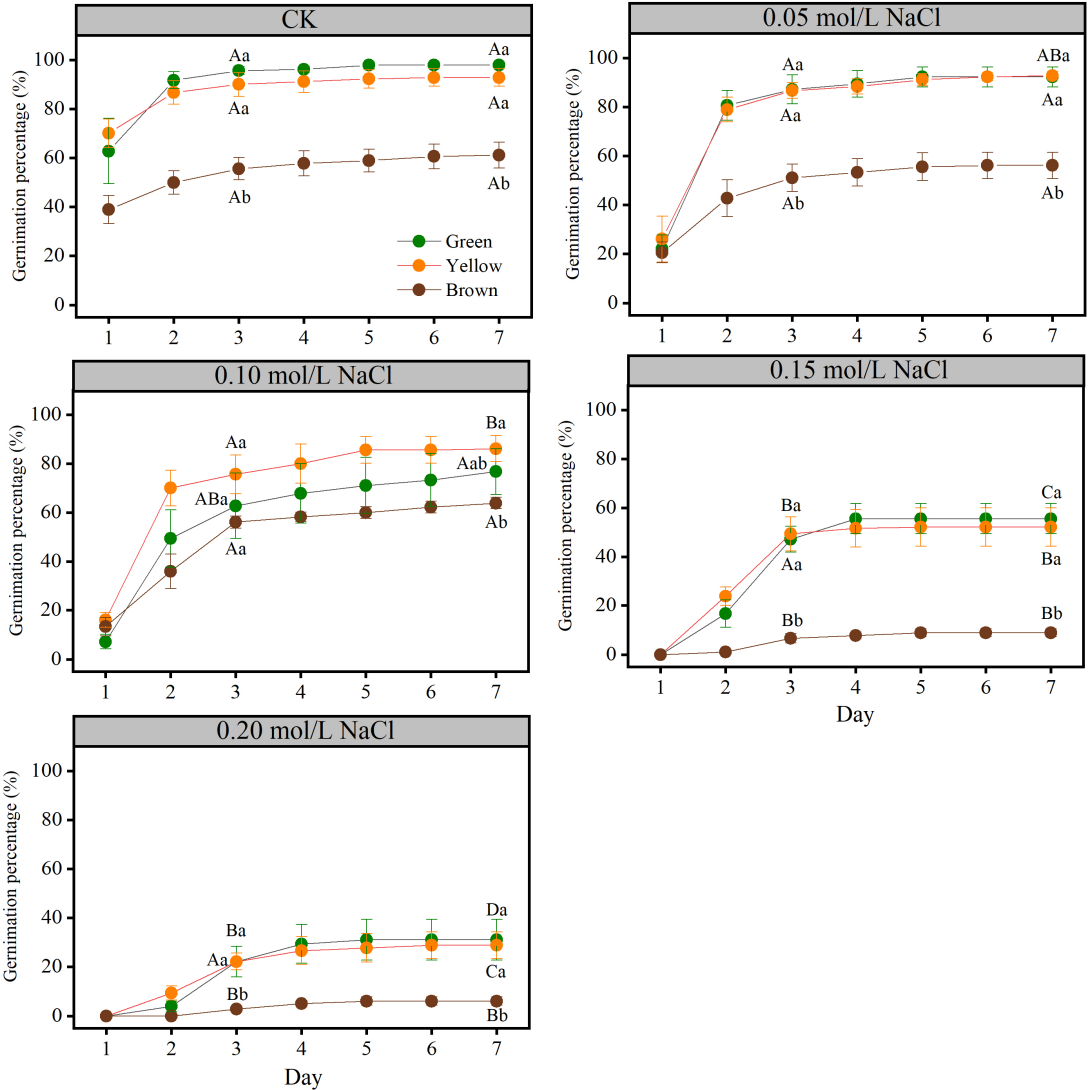
The seed germinability and final germination percentage of different color in different NaCl solution. Lowercase letters represent the difference among colors at the same NaCl solution, Capital letters represent the difference of the same color among different NaCl solution.

Seed color affected the germination parameters significantly (*P*< 0.05). The germinability and final germination percentage of yellow and green seeds were significantly higher than that of brown seeds (**Figure 1**), but they showed no significant differences between green and yellow seeds (*P* > 0.05).

**Figure 2:**
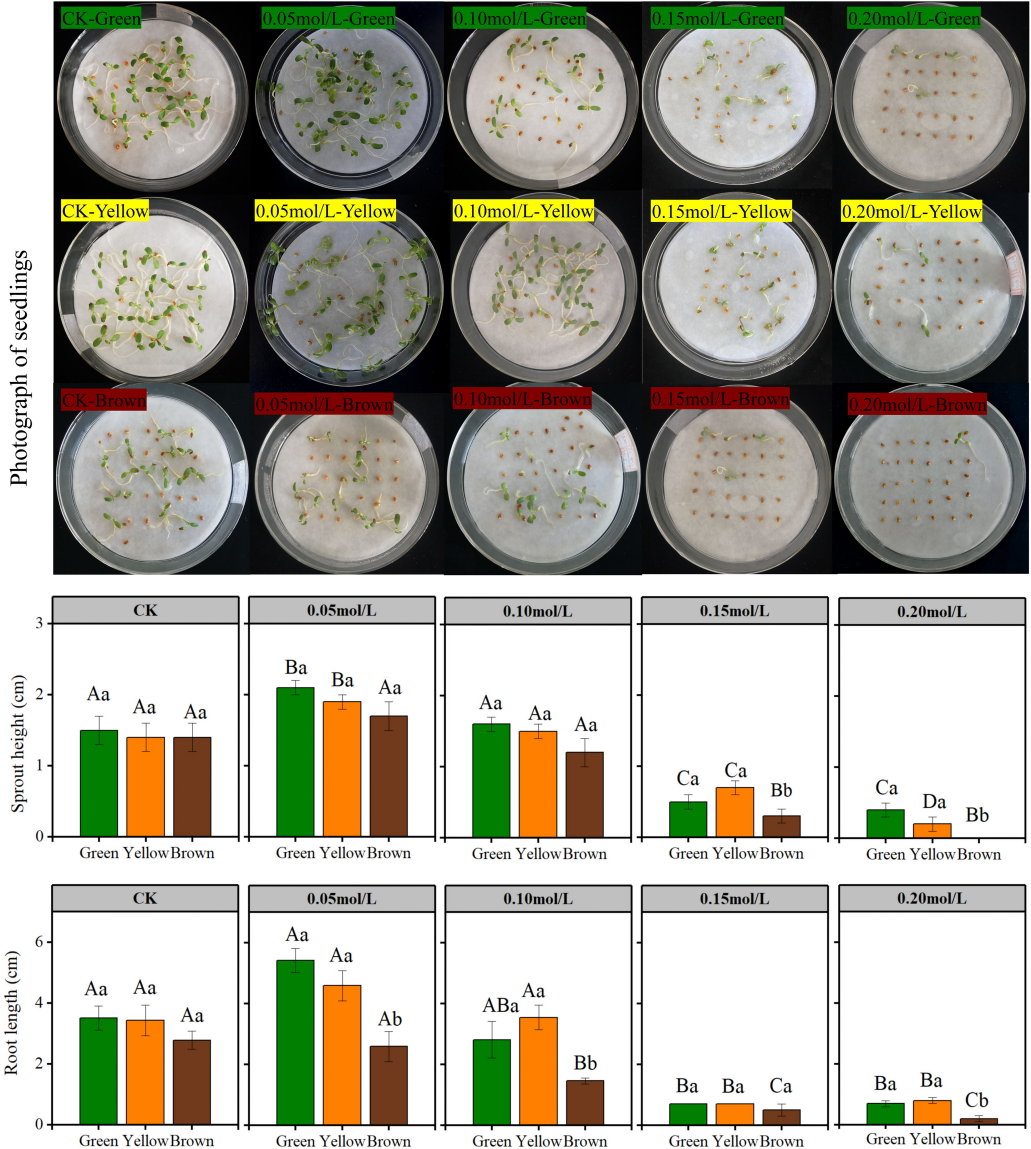
The seedling growth of different color in NaCl solution. Lowercase letters represent the difference among colors at the same NaCl solution, Capital letters represent the difference of the same color among different NaCl solution.

Salt stress had significant influence on the germination parameters (**Figures 1** and **2**). Generally, the germinability and final germination percentage decreased with the increase of salt stress. Take green seeds as an example, seed germinability and final germination percentage under CK (0.00 mol/L), 0.05 mol/L and 0.10 mol/L salt stress were not significantly different, but they were significantly higher than that under 0.15 mol/L and 0.20 mol/L salt stress (*P*< 0.05) (**Figure 1**).

**Figure 3:**
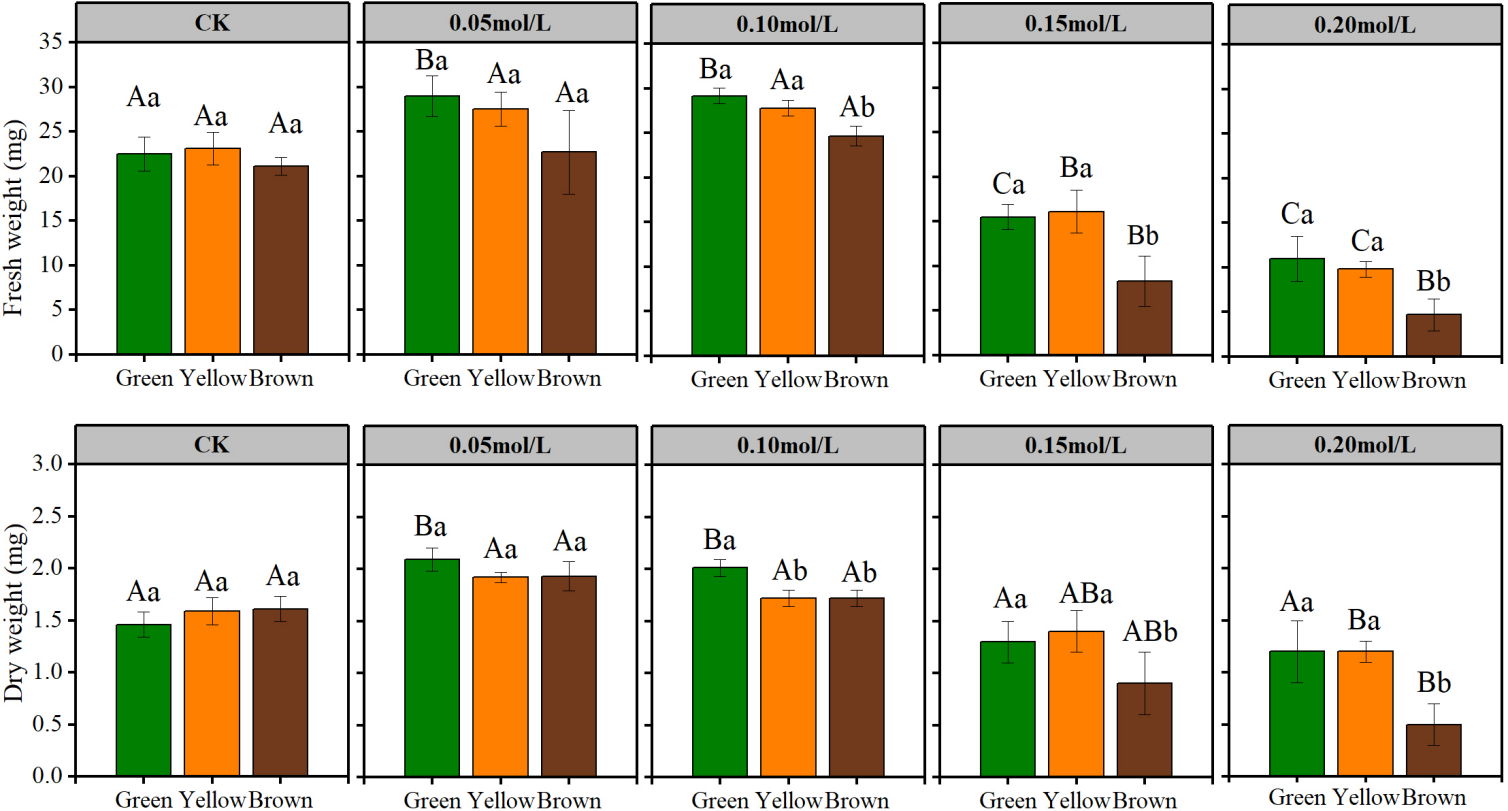
The fresh and dry weight of seedling in NaCl solution. Lowercase letters represent the difference among colors at the same NaCl solution, Capital letters represent the difference of the same color among different NaCl solution.

Seed color affected seedling growth significantly in high salt stress (**Figures 2** and **3**). In the treatment of 0.15 mol/L and 0.20 mol/L NaCl, the sprout height, root length, seedling fresh and dry weight of green and yellow seeds were significantly higher than that of brown seeds (**Figures 2** and **3**). Particularly, most brown seeds had no sprout under high salt stress.

Salt stress had great influence on seedling growth (**Figures 2** and **3**). The sprout height, root length, seedling weight were highest under 0.05mol/L salt stress, while these growth indexes were lowest under 0.20 mol/L salt stress (**Figures 2** and **3**).

### 3.2 Permeability properties of seed coat

**Figure 4:**
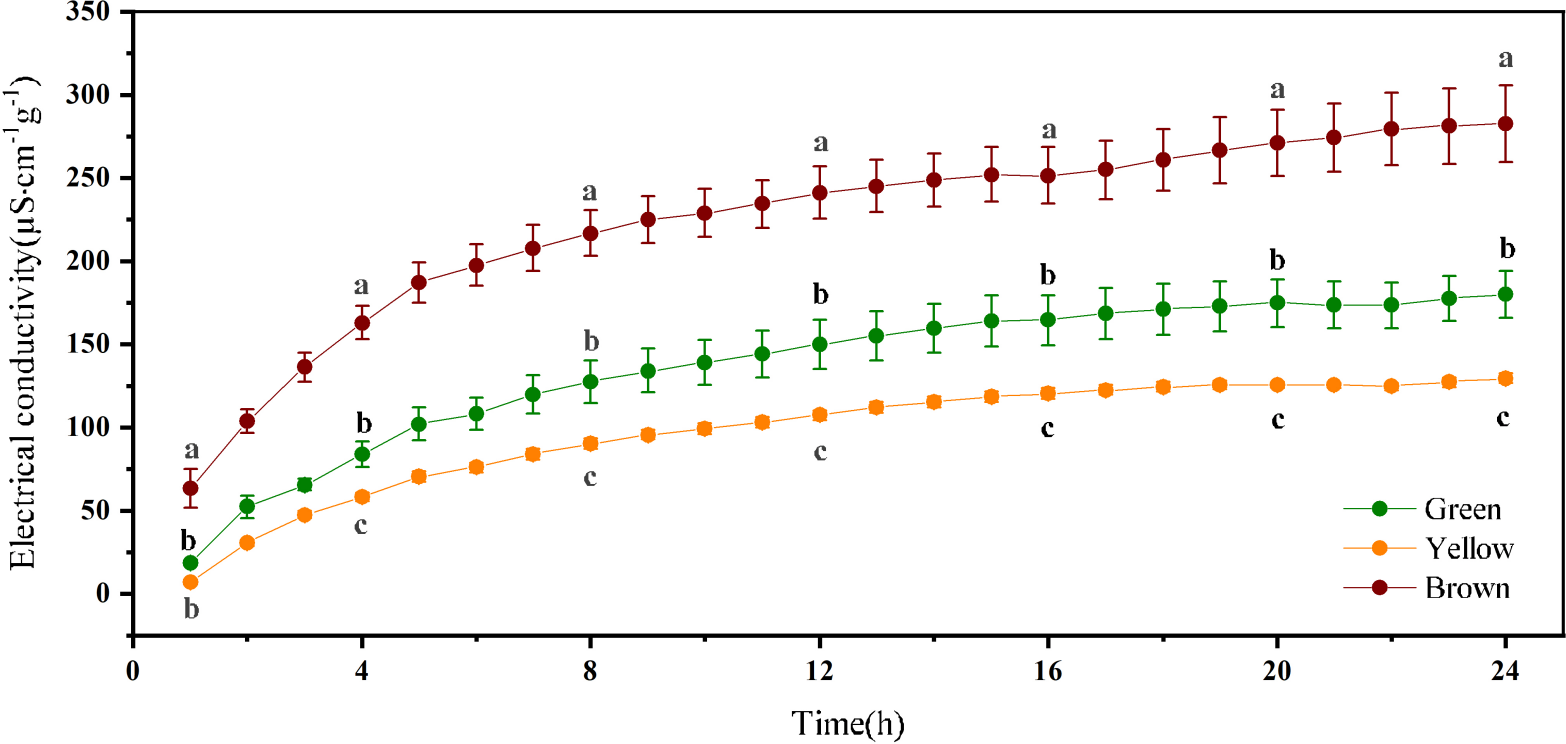
Electrical conductivity of alfalfa seeds of different color. Different lowercase letters represent the difference among three colors at the same time.

The variation of EC over time is consistent among the three-color seeds (**Figure 4**). The leachate values for EC increased fast within 4h, and gradually became stable after soaking 12 h. The leachate values for EC varied with seed color significantly. The brown seeds had the highest EC, and the EC of green seeds were larger than yellow seeds (*P*< 0.05).

**Figure 5:**
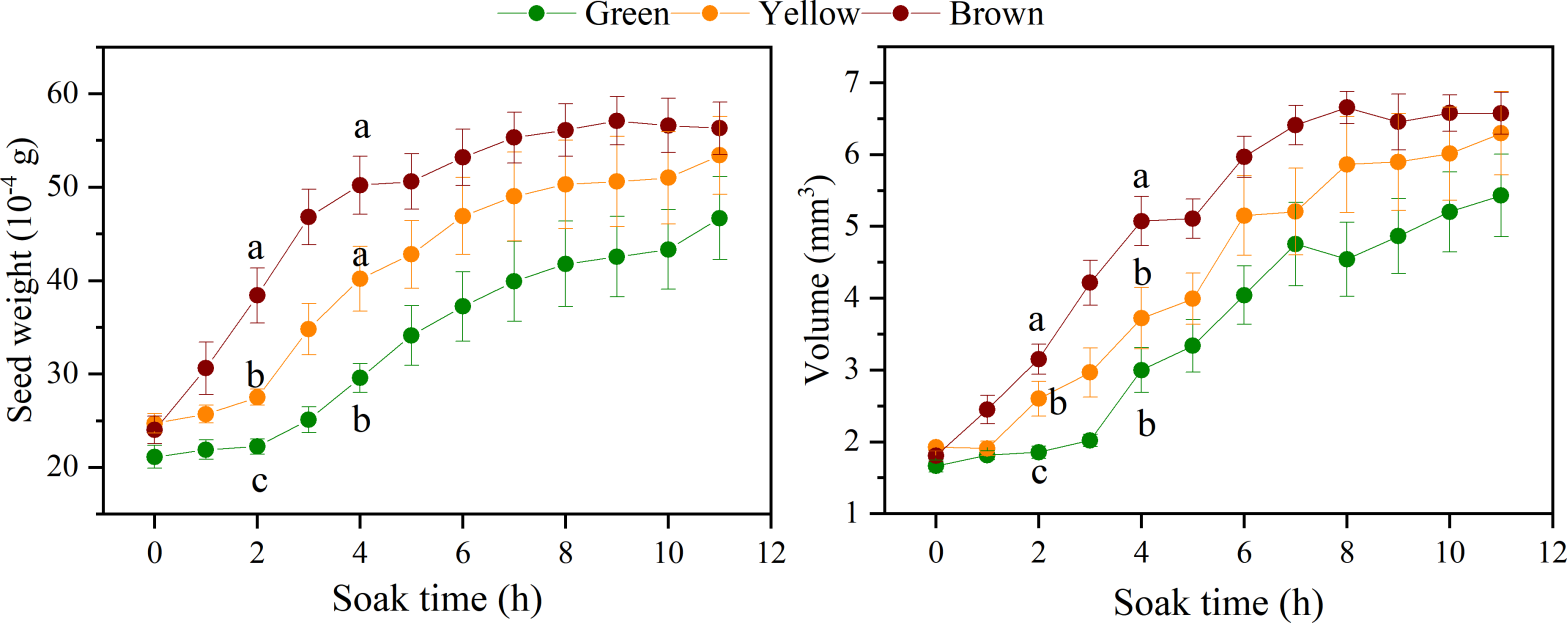
The change of seed weight and volume of different color over soaking time.

Changes in seed weight and volume reflected the water uptake capacity. The seed weight and volume changed rapidly within 4h after soaking, and then became stable after the 8 h (**Figure 5**). The seed weight and volume of three colors were significantly different at the 2 h, shown as brown seeds > yellow seeds > green seeds (*P<* 0.05), and the difference among the three colors became not significant over the soaking time. The weight and volume of brown seeds increased fast than that of green and yellow seeds, especially in the first 4h, the increase rate of three colors presented as brown seeds (109.2% and 181.2%) > yellow seeds (81.2% and 114.0%) > green seeds (45.2% and 81.7%).

**Figure 6:**
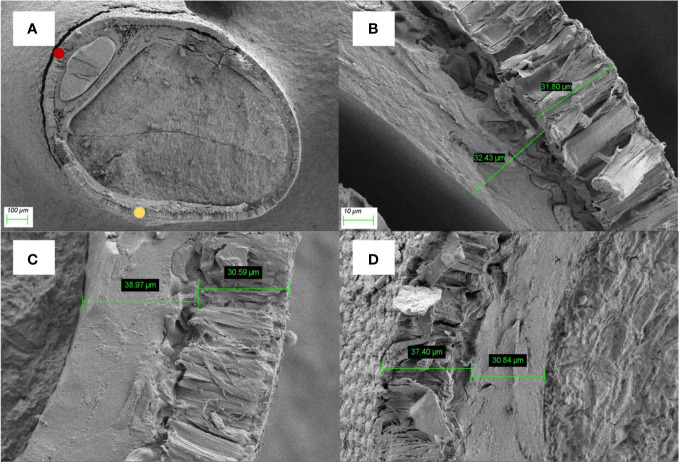
Graphs of the seed coats of alfalfa: **(A)** an aerial view of seed, bar=100μm; **(B)** Green seeds, bar=10μm; **(C)** Yellow seeds, bar=10μm; **(D)** Brown seeds, bar=10μm. The yellow dot represents the back of seed and the yellow dot represents the ventral of the seed.

The thickness of the palisade layer in the ventral of seed was shown that brown seeds (29.95 ± 1.33μm) > green seeds (28.96 ± 0.53μm)> yellow seeds (28.92 ± 1.38μm), but the differences among the three colors were not significant (*P* > 0.05) (**Figure 6**). As for the palisade layer in the back of seed, there was no significant difference either (brown 40.46 ± 2.46μm > yellow 35.39 ± 2.87μm> green 34.14 ± 1.86μm) (**Table 2**).

**Table 2:** Seed coat thickness of different color.

Coat color	palisade layer in the ventral of seed (μm)	palisade layer in the back of seed (μm)
Brown	29.95 ± 1.33a	40.46 ± 2.46a
Yellow	28.92 ± 1.38a	35.39 ± 2.87a
Green	28.96 ± 0.53a	34.14 ± 1.86a

Averages followed by the same letter within a column are not significantly different at the level P< 0.05. n=7 for the ventral of seed, n=8 for the back of seed.

### 3.3 Endogenous hormone content

**Figure 7:**
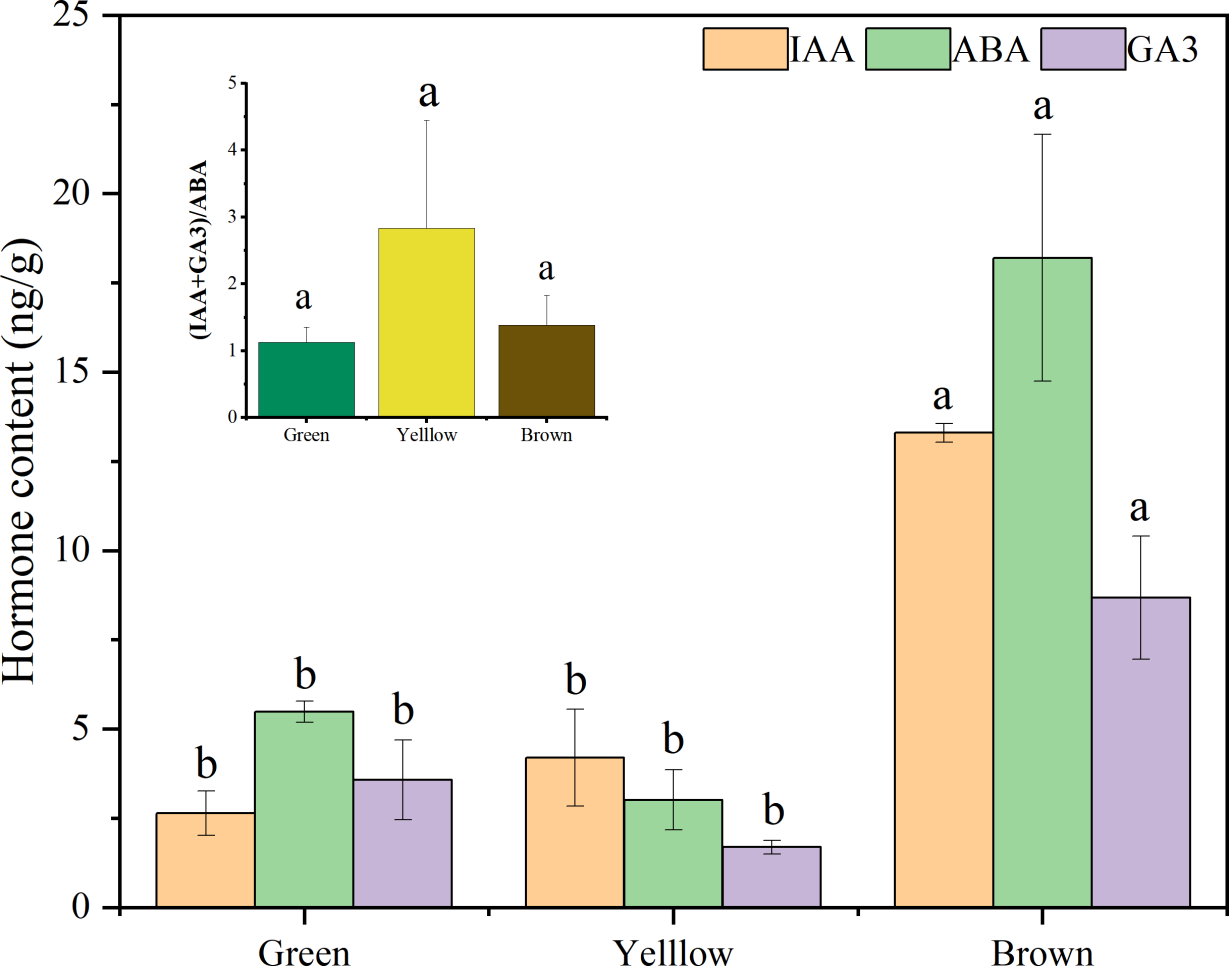
The hormone content in alfalfa seeds of different color. Lowercase letters represent the significant difference of same hormone among three colors, capital letters represent the difference of same color among hormone types at *P<* 0.05.

The hormone content remarkably varied with seed color and hormone type (**Figure 7**). The content of IAA and GA3 in brown seeds were significantly higher than those in yellow and green seeds (*P<* 0.05), but there was no significant difference between yellow and green seeds (*P >* 0.05). The content of ABA showed a significant difference among the three colors (brown: 21.02ng/g > green: 6.00ng/g > yellow: 2.92ng/g) (*P<* 0.05). The value of (IAA+GA3)/ABA in yellow seeds was larger than that in green and brown seeds, but it showed no significant difference among the three colors (*P >* 0.05).

## 4 Discussion

### 4.1 Effects of seed color on salt resistance of alfalfa

Due to the long flowering episode, the maturity of seeds in one lot is quite different at the same time and the seed coat acquires its own color along maturity (Lv et al., 2021). In our study, the yellow alfalfa seeds comprised the major part of the seed lot, and yellow seeds had higher vigor and germination parameters (germinability and final germination percentage), so it is recommended that yellow seeds could be the first choice in seed screening and planting for marginal land. More importantly, we found that the germination parameters and seedling growth indicators (sprout and root length, seedling weight) of brown seeds were not significantly different with control (no salt stress) in low salt stress (0.05 mol/L and 0.10mol/L NaCl) (**Figure 1**), but the germination ability of brown seeds fell sharply under high salt stress (0.20 mol/L NaCl), i.e. the germination rate decreased from 63.9% to 7.3%. However, the germination parameters of green and yellow seeds decreased with the aggravation of salt stress, and the seedling growth indicators under low salt stress were greater than control and high salt stress. Compared with green and yellow seeds, brown seeds were more sensitive to salt stress. And not as expected, the seed germination ability and seedling growth of brown seeds were lower than green and yellow seeds under different salt stress. Therefore, contrary to the first hypothesis, salt resistance of alfalfa seeds did not increase with the deepen of seed color. In fact, the salt resistance of brown seeds was significantly lower than that of green and yellow seeds.

### 4.2 Effects of seed color on seed traits

Seed germination and seedling establishment was affected by seed vigor, while seed color is a morphological trait associated with seed vigor (Murray et al., 2004; Moles et al., 2005; Yang et al., 2008). In this study, the results of electrical conductivity showed that the vigor of yellow alfalfa seeds was highest, and the vigor of brown alfalfa seeds was lowest. These findings were consistent with the results of red clover which reported that yellow seeds had higher vigor than brown and purple seeds (Atis et al., 2011).

Seed coat permeability limits the water uptake, which is the primary process of seed germination (Bewley et al., 2013). Previous studies showed that seed color leads to differences in water uptake (Ertekin and Kirdar, 2010). In our study, brown alfalfa seeds had a higher water uptake rate than green and yellow seeds at the early stage (**Figure 5**). Previous research reported that the cuticle and palisade layers in the seed coat caused the impermeability, so a thicker cuticle and palisade layers may reduce the rate of water uptake. However, the results of our study showed that the seed coat thickness of alfalfa was not significantly different among colors. It indicated that seed coat thickness may play little role in restricting the water from entering the seed. In addition, we found that seeds absorb water quickly did not germinate quickly and had a lower germinability and germination percentage (**Figure 1**). Our results were different from those of Zhang et al. (2008), they found that yellow rape seeds had rapid water uptake than black seeds. But our results were similar with the research of Atis et al. (2011), they observed that brown seeds of red clover had higher water uptake rate and lower germination percentage. The higher water uptake capability and lower germination of brown seeds may be caused by the damage of the seed coat structure. It has been reported that seed coat structure gradually breaks with the aging of seeds (Srii et al., 2022). Thus, the brown seeds lose membrane integrity, and they were prone to imbibitional injury, resulting in excessive water absorption and less oxygen (Mcdonald et al., 1988; Asiedu and Powell, 1998; Yang et al., 2008; Doria et al., 2019). The results that brown seed showed a faster change rate in electrical conductivity compared with green and yellow seed also exemplified that reason.

Plant hormones regulate seed germination and dormancy (Graeber et al., 2012, Jordan et al., 2017). In this study, the content of ABA, GA3, and IAA remarkably varied with seed color. The hormone content in brown seeds was significantly higher than those in yellow and green seeds, and noteworthy, the content of ABA in brown seeds was significantly higher than IAA and GA3. In the early stage of seed development, a higher concentration of growth promoting hormones (IAA and GA3) is required to promote the formation of the embryo body (Miransari and Smith, 2014); and then with the maturation of the seed, the content of ABA increased (Andrade et al., 2008; Matilla and Matilla-Vazquez, 2008). The difference in hormone content resulted in the lowest germination index of brown seeds (**Figure 1**). In addition to this, seed germination is not only related to the absolute content of hormones but also depends on the balance between various hormones (Baskin et al., 2000; Wang et al., 2018). The ratio between promoter and inhibitor [(IAA+GA3)/ABA] in yellow seeds was larger than that in green and brown seeds. Therefore, although the hormone content of yellow seeds including IAA and GA3 is lower than that of brown seeds, the germination parameter and seedling growth of yellow seeds are higher than brown seeds under salt stress. The findings of seed germination in our study were following those of Atis et al. (2011). They also reported that the brown-colored seeds had the lowest germination percentage (58%), whereas the yellow-colored seeds had the highest germination percentage (99%). It confirmed that both the content and the balance between IAA+GA3 and ABA played important role in the germination of seeds with different colors. Thus, the results of seed water uptake, seed coat thickness and hormone content supported the second hypothesis.

Based on the results, the green and yellow seeds germinated at a high rate (more than 95%) under normal conditions, while the brown seeds had a significantly lower germination rate (less than 50%), this difference may also associated with seed dormancy. According to Baskin and Baskin (2004), the green and yellow alfalfa seeds that used in the study was non-dormant, while brown alfalfa seed was more influenced by physiological dormancy (Type 1, non-deep PD). The balance of ABA and GA regulates the onset, maintenance and termination of physiological dormancy (Baskin et al., 2000). The change degree of ABA and GA over time may be different among three colors, which might influence the germination process and stress resistance. The water-impermeable palisade layers in seed coat hinder the water uptake, and then reduce the germination percentage. On the other hand, the broken seed coat may result in a very quick water absorption, which in turn lead to imbibition injury. Therefore, seed coat integrity and structure might have potential function in stress resistance. Except that the seed coat thickness was measured in a relatively small sample size, our study only examined the initial hormone content and seed coat thickness of one alfalfa cultivar. Further studies are needed to examine the hormone change and seed coat structure during the germination in alfalfa seeds of different dormancy type.

Despite the limitation, our study demonstrated the importance of the seed color with respect to the screening of seeds with high salt resistance and provided new insights for seed adaptability in terms of seed heteromorphism.

## 5 Conclusion

This study looks into the seed salt resistance by systematically evaluating the performance of seed germination and seedling growth under salt stress, and estimating the seed traits. Our study founded that seed color had significant influence on salt resistance of alfalfa seeds. Green and yellow alfalfa seeds had greater germination ability and stronger seedling growth than brown seeds in different NaCl solution, it indicated that green and yellow seeds had higher salt resistance than brown seeds. Besides, the electrical conductivity and seed water uptake rate of brown seeds were greater than green and brown seeds, but the coat thickness was not influenced by seed color. The results indicated that seed coat thickness may play little role in regulating salt resistance by altering permeability. Brown seeds had higher IAA, GA3 and ABA content, but lower (IAA+GA3)/ABA than yellow and green seeds. The endogenous hormone played more important role in affecting the salt resistance.

## Data availability statement

The raw data supporting the conclusions of this article will be made available by the authors, without undue reservation.

## Author contributions

JX conceived the study and wrote the original draft, GJ and YL took the laboratory job, HS and XL participated in the review and revision of the original draft, and LH supervised the experiment. All authors contributed to the article and approved the submitted version.
